# A focus on caging radicals: a catch-and-release mechanism for BODIPY photocages

**DOI:** 10.1039/d6sc90197b

**Published:** 2026-10-19

**Authors:** Jiarong Wu, Jake L. Greenfield

**Affiliations:** a EaStCHEM, School of Chemistry, University of St Andrews St Andrews KY16 9ST UK jlg28@st-andrews.ac.uk

## Abstract

Photocages enable the on-demand release of a payload in a closed chemical system upon irradiation with a suitable wavelength of light. Most photocages are described as releasing their payload through heterolytic cleavage of the photolabile bond, producing anionic and cationic fragments. Homolytic cleavage to form radicals is less commonly exploited as they react more indiscriminately and can generate unintended products. Poryvai and co-workers (A. Poryvai, A. Vasiļevska, K. Bangievská, J. Tarábek, S. Gerber-Lemaire and T. Slanina, *Chem. Sci.*, 2026, **17**, 11430, https://doi.org/10.1039/D6SC01848C) show how homolytic bond scission can instead be used to achieve a catch-and-release mechanism for radical payloads. Using a BODIPY-based photocage that responds to green light (525 nm), they demonstrate the capture of TEMPO and its subsequent release on demand. This behaviour was also applied to radical-based polymerisation. This work establishes design principles for the catch-and-release mechanism, and draws attention back to the often overlooked homolytic pathway in photocage chemistry.

## Abstract 2

Focusing on the often-overlooked homolytic bond-cleavage mechanism of photocages, Poryvai *et al.* (*Chem. Sci.*, 2026, https://doi.org/10.1039/D6SC01848C) exploit this pathway to demonstrate the on-demand catch-and-release of radical payloads and apply this platform to radical polymerisation reactions.

## Graphical abstract

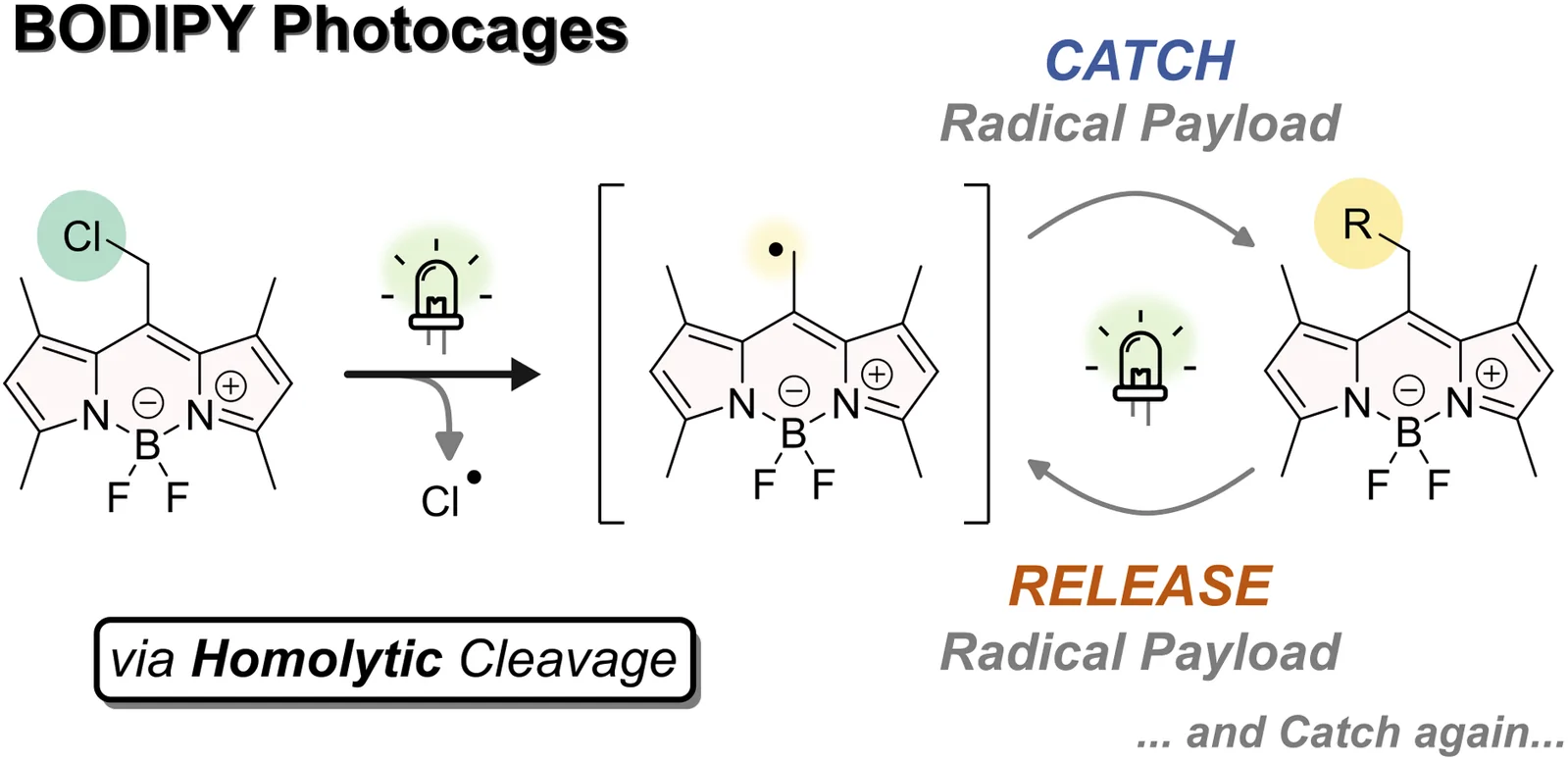

A central aim of synthetic chemistry is to modify molecules selectively.^1,2^ Protecting groups provide one means of achieving this by temporarily masking a specific functional group to prevent unwanted, or incompatible reactivities.^3,4^ Most protecting-group strategies require chemical reagents to be introduced to the system for the protection and deprotection steps. While working with an open chemical system is synonymous with synthetic chemistry, many applications require an unmasking of a functionality in a closed chemical system (*vide infra*). Photolabile protecting groups,^5^ commonly termed photocages,^6,7^ can be used for such cases.

Photocages release a payload upon irradiation with a suitable wavelength of light.^8^ The use of light as a trigger is advantageous as it provides high spatiotemporal control over the payload release,^9^ together with selectivity arising from the electronic properties of the chromophore.^5,10^ This enables a molecule to be deployed in a deactivated state and subsequently activated, on demand, in a closed chemical system. Photocages have found use in a variety of fields.^6^ In chemical biology,^7,10^ for example, a warhead can be masked until irradiation releases it at the desired site, potentially reducing off-target effects. In supramolecular chemistry, a subcomponent can remain masked until photoirradiation triggers its release, enabling the on-demand assembly of molecular capsules.^11^

Unlike photoswitches, which can reversibly interconvert between two or more states in response to light, photocages typically undergo cleavage of a covalent bond that is practically irreversible.^12^ This cleavage can occur either heterolytically or homolytically. For heterolytic processes (Fig. 1(i)), both bonding electrons remain with one fragment, generating an anionic species and a cationic counterpart. For homolytic processes (Fig. 1(ii)), each fragment retains an electron from the cleaved bond, producing two radicals. Homolytic cleavage is generally considered less desirable because the resulting radicals are less selective in their subsequent reactions, reacting with other compounds in the system, and potentially generating unintended products or off-target effects.

**Fig. 1:**
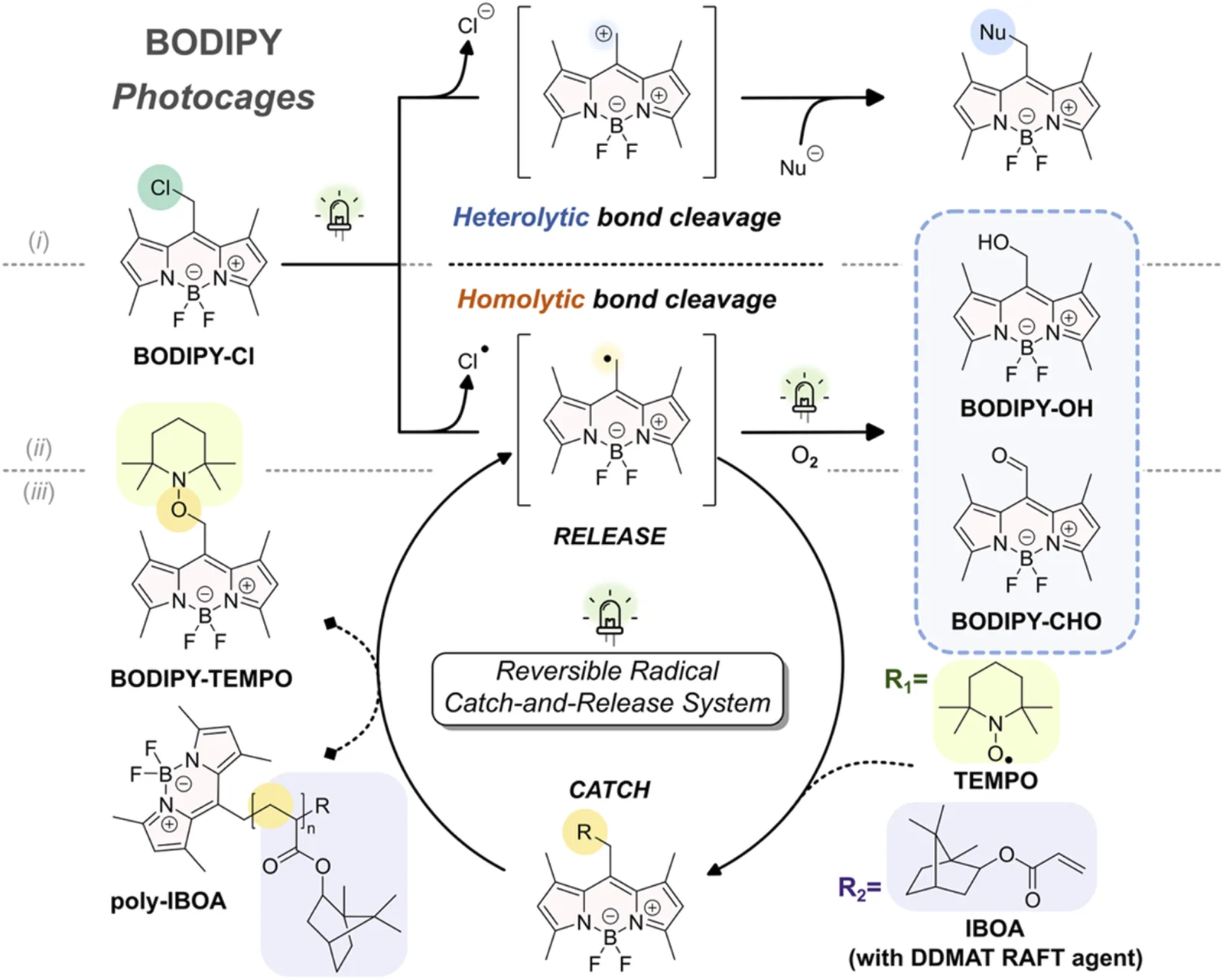
Schematic illustration of the green-light-induced catch-and-release mechanism for BODIPY photocages. Cleavage of the photolabile bond can be achieved either (i) heterolytically, or (ii) homolytically. For the homolytic pathway (ii), a BODIPY-centered radical is formed which can undergo reversible catch-and-release cycles (iii). TEMPO (green) and IBOA (purple) payloads were shown to be captured and released, with the latter being used in the polymerization of IBOA to achieve poly-IBOA. It is important to note here that the release step does not regenerate BODIPY-Cl; the process is therefore reversible at the level of BODIPY–payload bond formation and cleavage steps, but it is not a closed molecular cycle that restores the initial BODIPY-Cl cage.

In a recently published article (https://doi.org/10.1039/D6SC01848C),^13^ Poryvai and co-workers draw attention back to the often overlooked homolytic process. By biasing the system towards a homolytic cleavage process, they were able to demonstrate light-induced capture of a radical payload followed by its release under green-light irradiation (Fig. 1).

Specifically, irradiating the starting BODIPY-Cl with green light (525 nm) generated a BODIPY-centred radical intermediate. This radical was subsequently caught by TEMPO to form the BODIPY-TEMPO adduct. Continued irradiation of the system results in further homolytic cleavage, releasing the TEMPO radical and regenerating the BODIPY-centred radical. Under aerated conditions, the BODIPY radical reacts to form BODIPY-OH and BODIPY-CHO as products of the radical pathway.

This so-called “catch-and-release” mechanism provides a platform whereby different payloads can be installed, at a late stage, onto the BODIPY core under light-irradiation, and subsequently deployed (Fig. 1(iii)). As each catching-step involves the release of the currently attached payload, a degree of cyclability can be achieved. This was neatly shown in the context of free-radical and Reversible Addition–Fragmentation chain-Transfer (RAFT) polymerization of isobornyl acrylate (IBOA), using BODIPY-Cl as a type I photoinitiator.^14,15^ Increasing the irradiation time increased monomer conversion, and the formation of BODIPY-RAFT intermediates were further identified in the system, suggesting that the catch-and-release mechanism operates over the course of the polymerisation.

The authors also neatly examined how modification of the BODIPY chromophore affects the catch-and-release mechanism. Notably, the efficiency of the homolytic cleavage was reduced following iodination of the BODIPY core. This modification was observed to increase the efficiency of triplet state formation, attributed to enhanced intersystem crossing *via* the heavy-atom effect. Conversely, efficient homolysis was observed for systems displaying a high fluorescence quantum yield, consistent with cleavage occurring in the singlet excited state. Fluorescence measurements therefore provide useful indicators of the balance between the homolytic and heterolytic cleavage pathways.

The identity of the payload also offers another potential handle for controlling the bias between the two cleavage mechanisms. A broader and systematic comparison of payloads could establish whether their electronic properties can be used to favour homolytic capture but heterolytic release. Such behaviour could retain the practical advantage of late-stage radical trapping while reducing radical generation during uncaging, or provide orthogonal reactivity between the photoproducts.

A study into the wavelength dependence of the catch-and-release mechanism may also reveal additional ways to tune the efficiency of the homolytic pathway. It is often assumed that the absorption maximum of a chromophore will also identify the wavelength at which its photochemical reaction proceeds most efficiently.^16^ Wavelength-dependent studies by Barner-Kowollik and co-workers have shown that absorptivity and photochemical reactivity frequently do not coincide;^17^ in many systems, the optimum reaction wavelength is shifted towards the red edge of the absorption spectrum.^16,18^

In particular, recent wavelength-dependent studies on a germanium-based photoinitiator,^19^ Ivocerin, have shown that photochemical action spectra may diverge markedly from absorption spectra because both radical-generation probability and the persistence of the radical vary with excitation wavelength. Applying a similar combination of action plots and time-resolved spectroscopy to BODIPY photocages could determine whether the excitation wavelength alters the efficiency of the singlet-associated homolysis, the lifetime and recombination of the resulting radical pair, or competition with heterolytic pathways.

Finally, while the system reported by Poryvai reaches impressive yields of up to 95%, it is not fully reversible. A fully reversible photocaging and decaging strategy, with selectivity over the payload, would be a valuable broader goal for the field as it would allow a released payload to be recaptured. If the payload were a drug, for example, it could be released long enough to exert its intended effect, and any remaining active material could then be re-caged. The recaptured species can then be cleared from the system, potentially further reducing off-target effects. Developing such a system represents an interesting challenge for the field of photocages and molecular photoswitches.

## Author contributions

Both J. W. and J. L. G. prepared and edited the initial draft. J. L. G. acquired funding and supervised the work.

## Conflicts of interest

There are no conflicts to declare.

## Data Availability

No primary research results, software or code have been included, and no new data were generated or analysed as part of this focus article.
